# Update on Genitourinary Syndrome of Menopause: A Scoping Review of a Tailored Treatment-Based Approach

**DOI:** 10.3390/life14111504

**Published:** 2024-11-19

**Authors:** Ilaria Cuccu, Tullio Golia D’Augè, Ilaria Firulli, Emanuele De Angelis, Giovanni Buzzaccarini, Ottavia D’Oria, Aris Raad Besharat, Donatella Caserta, Giorgio Bogani, Ludovico Muzii, Violante Di Donato, Andrea Giannini

**Affiliations:** 1 Department of Maternal and Child Health and Urological Sciences, Sapienza University of Rome, Policlinico Umberto I, Viale del Policlinico 155, 00161 Rome, Italy; tullio.goliadauge@uniroma1.it (T.G.D.); ilaria.firulli@uniroma1.it (I.F.); emanuele.deangelis@uniroma1.it (E.D.A.); ludovico.muzii@uniroma1.it (L.M.); violante.didonato@uniroma1.it (V.D.D.); 2 Department of Obstetrics and Gynaecology, IRCCS San Raffaele Scientific Institute, Vita-Salute San Raffaele University, Via Olgettina 58-60, 20132 Milan, Italy; giovanni.buzzaccarini@gmail.com; 3 Department of Medical and Surgical Sciences and Translational Medicine, PhD Course in “Translational Medicine and Oncology”, Sapienza University of Rome, Viale dell’Università, 37, 00185 Rome, Italy; ottaviadr@gmail.com (O.D.); besharataris@gmail.com (A.R.B.); andrea.giannini@uniroma1.it (A.G.); 4 Obstetrics and Gynecological Unit, Department of Woman’s and Child’s Health, San Camillo-Forlanini Hospital, 00152 Rome, Italy; 5 Gynecology Division, Department of Medical and Surgical Sciences and Translational Medicine, Sant’Andrea University Hospital, Sapienza University of Rome, Via di Grottarossa 1035, 00189 Rome, Italy; donatella.caserta@uniroma1.it; 6 Department of Gynecologic Oncology, IRCCS National Cancer Institute, Via Giacomo Venezian 1, 20133 Milan, Italy; boganigiorgio@gmail.com

**Keywords:** genitourinary symptoms, vulvovaginal atrophy, laser CO_2_, hyaluronic acid, estrogen, DHEA, ospemifene, menopause

## Abstract

This scoping review explores the therapeutic strategies available for managing genitourinary syndrome of menopause (GSM), a condition often underdiagnosed and undertreated despite significantly affecting women’s quality of life. GSM results from decreased estrogen levels during menopause, leading to a range of symptoms including vulvovaginal atrophy and urinary tract issues. Material and Methods: we screened the literature for original studies with “menopause”, “hormonal therapy”, “vulvovaginal atrophy”, “urinary incontinence”, “urinary infections”, “genitourinary syndrome”. Results: A total of 451 relevant articles were retrieved. After screening, 19 articles were included in this scoping review. Discussion: First-line treatments typically include lubricants and moisturizers for short-term symptom relief, while unresolved or severe cases may warrant hormonal treatment. Topical hormonal treatments often have fewer side effects than systemic alternatives. Special attention is given to selective estrogen receptor modulators like ospemifene and steroid hormones like dehydroepiandrosterone (DHEA), which have shown beneficial effects on GSM symptoms. Moreover, innovative therapeutic approaches, such as laser treatment, are discussed in the context of their efficacy and accessibility. The safety of GSM hormonal therapy in women with a history or risk of cancer is also addressed, noting the need for more definitive research in this area. While there is a growing demand for tailored therapy, this scoping review emphasizes the importance of effective communication and counseling to allow women to make informed decisions about their treatment. Overall, this review underscores the need for increased awareness and further research into effective treatment options for GSM.

## 1. Introduction

The genitourinary syndrome of menopause (GSM) is a highly prevalent condition, affecting more than half of menopausal women. Research indicates that approximately 65% of women experience symptoms within the first year of menopause, with this number increasing to around 85% after six years [1]. In 2014, the North American Menopause Society (NAMS) and the International Society for the Study of Women’s Sexual Health formally adopted the term GSM, which encompasses the signs and symptoms associated with menopause that specifically affect the genitourinary tract [1]. This chronic and progressive condition presents with a wide range of symptoms, though no definitive set of criteria has been established for diagnosis. The diagnostic approach generally relies on a combination of medical history, reported symptoms and physical examination. The decline in estrogen levels during menopause triggers changes and dysfunctions in the female genital system, leading to various complications such as pain, vaginal dryness, urinary incontinence, overactive bladder, dysuria, urinary tract infections, dyspareunia and other related issues [2].

The inevitable consequence of GSM is a substantial impact on quality of life, affecting psychological well-being, interpersonal relationships, professional life and sexual experiences. Nearly all cases involve vaginal atrophy, often signaled by a vaginal pH above 5. Unfortunately, GSM is frequently underdiagnosed, as its symptoms are often mistakenly attributed to normal aging processes, leading many women to endure these discomforts in silence [3]. Without timely intervention, the risk of complications, including recurrent urinary tract infections, meatal stenosis, urethral prolapse or atrophy, and the formation of urethral polyps, increases. Various treatment options, both hormonal and non-hormonal, are available to alleviate severe symptoms and address vaginal atrophy [4,5,6,7,8,9]. This review aims to summarize the current treatment recommendations for GSM, incorporating the latest scientific evidence, while highlighting the distinct characteristics of each therapeutic approach to support personalized care in clinical practice.

## 2. Materials and Methods

The search was conducted by different researchers independently, evaluating several independent databases (MEDLINE, EMBASE, Global Health, Cochrane Database of Systematic Reviews, Cochrane Central Register of Controlled Trials, Cochrane Methodology Register) to find all possible relevant trials. The keywords used to find relevant articles were “menopause”, “hormonal therapy”, “vulvovaginal atrophy”, “urinary incontinence”, “urinary infections”, “genitourinary syndrome”. Key criteria for inclusion were as follows: (1) full articles in English language; (2) original studies concerning genitourinary syndrome in menopause; (3) studies comparing different treatment for GSM. All articles were screened using the keywords by three independent authors ( I.F., E.D.A.). The search was conducted without date restriction until 31 January 2023.

All the studies screened through the inclusion and exclusion criteria were examined, and relevant data extracted for each paper.

The full text of these potentially eligible articles was retrieved and assessed for eligibility by another two independent review team members (I.C., T.G.D.). Any disagreement between them over the eligibility of some articles was resolved through discussion with an external collaborator (G.B). All the studies screened through the inclusion criteria were examined, and relevant data extracted for each paper. Two authors (I.C., T.G.D.) independently extracted data from articles about study characteristics and included populations, type of intervention and outcomes, using a pre-piloted standard form in order to ensure consistency. Due to the nature of the findings, we opted for a narrative scoping synthesis of the results from selected articles.

## 3. Results

**Figure 1:**
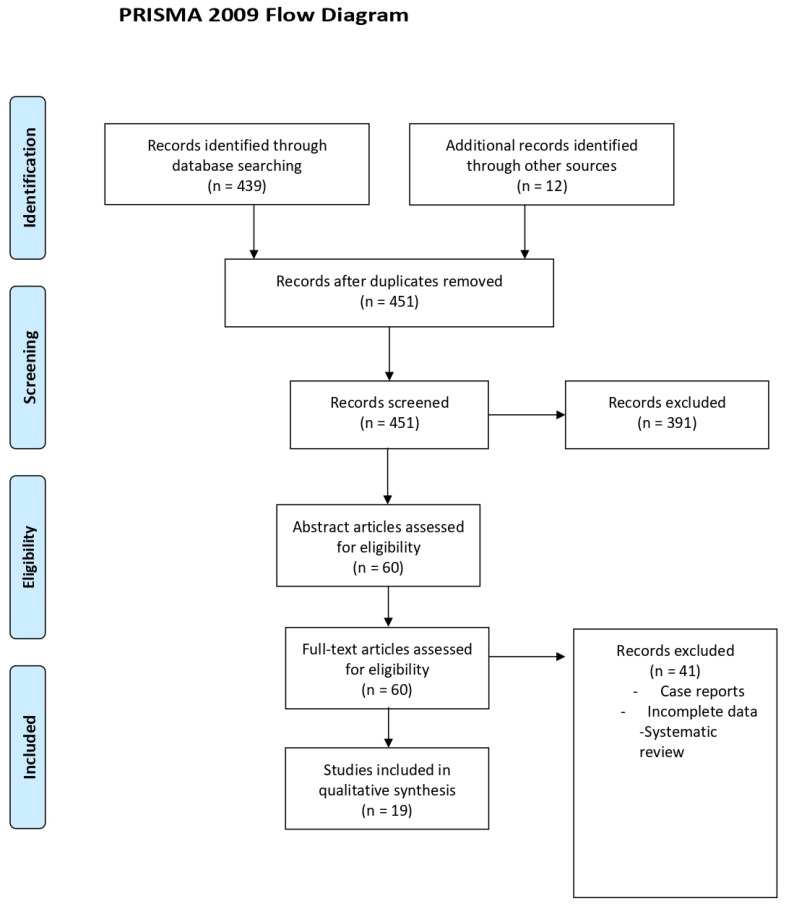
PRISMA flow diagram results of the search strategy [28].

A search of the MEDLINE (PubMed) database resulted in 451 relevant articles. The search was narrowed to articles between 2003 and 2023. A total of 60 materials were initially identified to be potentially relevant for the review. Finally, 19 articles were included, and they were found to match the inclusion criteria [9,10,11,12,13,14,15,16,17,18,19,20,21,22,23,24,25,26,27] (Figure 1). Excluded papers were non-English language, review papers, abstracts or poster presentations. For the purposes of this comprehensive review, we opted for a narrative presentation for results, see Table 1. However, we also decided to summarize principal treatment options for GSM, considering mechanisms of action, effects and application methods, as reported in Table 2.

## 4. Discussion

### 4.1. Vaginal Moisturizers and Lubricants

Vaginal therapies, such as moisturizers and lubricants made from water, silicone or oil-based substances, are recommended as the first-line pharmacological treatment for GSM by both the North American Menopause Society (NAMS) [29] and the International Menopause Society. These treatments have proven effective in alleviating symptoms [10], with vaginal lubricants and moisturizers being commonly used as primary interventions for GSM. They are generally considered safe and well-tolerated [30]. Various active compounds are available for topical use to treat symptoms such as vaginal dryness and dyspareunia by enhancing hydration and lubrication, making them a viable alternative to hormonal treatments. Menopause-associated hormonal changes reduce hyaluronic acid (HA) production, resulting in thinning of the vaginal epithelium, decreased blood flow, loss of tissue elasticity and symptoms such as dryness and discomfort [1]. HA plays a critical role in maintaining water balance and tissue integrity within the urogenital system. Thanks to their viscoelastic properties, HA-based products effectively relieve these symptoms. Lubricants and moisturizers—whether water, silicone, mineral or plant oil-based—serve a variety of applications. Water-based lubricants are the most widely accessible and tend to cause fewer genital side effects than those made from other ingredients [11].

Natural oils like olive, coconut and mineral oil can also be used for vaginal moisturization; however, further clinical trials are necessary as some studies suggest these oils may impair sperm function in vitro [31]. Vaginal lubricants are recommended to have a pH of approximately 4.5. The World Health Organization (WHO) advises that the osmolality of personal lubricants should not exceed 380 mOsm/kg to prevent epithelial damage, including mucosal irritation, tissue damage or cytotoxicity [32]. Vaginal moisturizers are recommended for women experiencing daily discomfort due to vaginal dryness, atrophic vaginitis or vulvar and vaginal atrophy (VVA), not just for use during sexual activity. These moisturizers are absorbed through the skin and offer longer-lasting effects than lubricants. Both products can be used alongside other GSM treatments. A study with 98 patients suffering from recurrent urinary tract infections (UTIs) showed that combination therapy with oral hyaluronic acid (HA), curcumin and quercetin was effective in reducing and preventing UTI recurrence and episodes of dysuria [10]. Additionally, a multicenter, randomized, controlled, open-label, parallel-group clinical trial conducted by Chen et al. in 2013 found that both HA vaginal gel and estriol cream significantly improved symptoms of vaginal dryness in postmenopausal women. Among the 144 patients enrolled, 84.44% of those using HA gel and 89.42% of those using estriol cream reported symptom improvement, with no statistically significant difference between the two treatments. This suggests that HA vaginal gel could be a suitable alternative to estrogen-based treatments for relieving vaginal dryness [33].

### 4.2. Estrogens and Progestins

For the treatment of GSM, hormone therapy using either estrogen alone or a combination of estrogen and progestin is commonly employed. These treatments can be administered orally or topically, with the distribution of estrogen and progesterone receptors being more concentrated in the upper vagina and less so at the vulvovaginal junction in the external genitalia [12]. The appropriate dosage and method of administration are determined by the severity and specific type of genitourinary symptoms. Both the North American Menopause Society (NAMS) and the International Menopause Society recommend vaginal therapy as the first-line pharmacologic treatment for GSM. Topical application is typically adequate for mild symptoms, whereas more severe cases may require oral administration [13].

Commonly prescribed estrogen options include conjugated equine estrogen (CEE), synthetic conjugated estrogens, micronized 17b-estradiol and ethinyl estradiol. Lower doses designed for vaginal use are available in formats such as creams, sustained-release rings, vaginal tablets and soft gels. Systemic administration can be delivered orally or through transdermal sprays or gels [13]. The effectiveness and safety of local estrogen treatments have been examined in numerous randomized trials, comparing different estrogen preparations to each other and to placebos [14,15,34,35]. These studies consistently show that estrogen improves genitourinary symptoms, such as dyspareunia and vaginal dryness, compared to placebo, with no significant differences among various estrogen formulations. As a result, low doses of estrogen are recommended over medium and high doses, with 4 μg being identified as the lowest safe and effective dose according to current literature [36].

For patients with moderate to severe GSM, combined or non-estrogenic systemic therapy with progestin may offer benefits. Suggested doses include oral conjugated equine estrogen (CEE) at 0.3 mg, oral 17b-estradiol at less than 0.5 mg or an estradiol patch at 0.025 mg, which typically take 6 to 8 weeks to relieve symptoms. While serum estrogen levels usually remain within the normal range, oral estrogen can cause side effects such as elevated inflammatory markers, coagulation factors, hypertriglyceridemia, dementia, coronary artery disease, gallstones and venous thromboembolism, particularly in women starting estrogen therapy before menopause or those over 60 years of age [37]. A large observational study with over 80,000 participants [38] indicated that women on oral therapy had a higher risk of venous thromboembolic events compared to those using transdermal therapy or no therapy. Prolonged systemic estrogen exposure also raises the risk of endometrial thickening, hyperplasia and cancer. For women with an intact uterus, combining progestin with estrogen is recommended, as progestin counteracts estrogen-induced receptor proliferation and lowers circulating luteinizing hormone levels by modulating hypothalamic-pituitary signaling [16]. Common progestogens include medroxyprogesterone acetate, norethindrone acetate and natural progesterone. Continuous regimens reduce the risk of endometrial cancer compared to sequential regimens, though no significant differences are observed between progestogen types or methods of administration [39]. The combination, dosage and duration of therapy should be individualized. Additionally, a large randomized trial [16] showed that combination therapy improves vasomotor symptoms and vaginal dryness, though side effects such as vaginal bleeding and breast tenderness may occur, with incidence decreasing to around 13% by the fifth year of treatment.

### 4.3. Ospemifene

Ospemifene is considered an effective treatment for vulvovaginal atrophy (VVA) in postmenopausal women who are not suitable candidates for estrogen therapy [13]. As a Selective Estrogen Receptor Modulator (SERM), it exhibits both agonist and antagonist properties, activating specific estrogen pathways while inhibiting others. Ospemifene is metabolized and cleared primarily by the liver [17,40]. VVA, which results from estrogen deficiency, presents with symptoms such as thinning of the vaginal epithelium, reduced lubrication, decreased lactobacilli colonization and an elevated vaginal pH. Studies show that after approximately one month of treatment with ospemifene, these symptoms improve, with thicker vaginal epithelium, increased epithelial cell proliferation and greater expression of estrogen receptor alpha (ERα) observed in treated patients compared to those not receiving therapy [18].

A meta-analysis by Di Donato et al., which included six randomized controlled trials, evaluated ospemifene’s efficacy over 12 and 52 weeks compared to placebo. The results indicated that a daily dose of 60 mg significantly improved both the morphological characteristics of the vaginal mucosa, such as a reduction in parabasal cells and an increase in superficial cells, as well as physiological features, including lower vaginal pH, reduced dyspareunia and improved sexual function [19,41].

At 12 weeks, the primary side effects of ospemifene included hot flashes and urinary tract infections; however, these adverse effects were not observed after 52 weeks of treatment. The incidence of headaches, deep vein thrombosis, coronary artery disease, cardiovascular events, severe adverse events, vaginal bleeding, endometrial cancer and breast cancer did not differ significantly from other therapies [18].

After 12 weeks of treatment with 60 mg of ospemifene, the percentage of patients with detrusor overactivity decreased from 39% to 13%, alongside a reduction in detrusor pressure and improvements in peak flow, initial voiding urge and maximum cystometric capacity [19]. In terms of safety outcomes—including endometrial health, breast health, thrombotic risk and adverse events—no significant differences were found between ospemifene 60 mg, 17b-estradiol 10 mg or estriol gel over a 12-week period. The reductions in vaginal pH and improvements in maturation values with ospemifene were similar to those achieved with 17b-estradiol 10 mg. Thus, ospemifene’s safety and efficacy can be considered comparable to, if not superior to, local estrogen therapy [41].

### 4.4. Dehydroepiandrosterone (DHEA)

Dehydroepiandrosterone (DHEA), also known as prasterone, is a steroid hormone crucial for converting cholesterol into androgens and estrogens [20]. From the age of 30–40 years, serum levels of androgens, including DHEA, begin to decline. Consequently androgen replacement therapy may have beneficial effects, such as improving local vaginal conditions and enhancing sexual quality of life [20]. This is due to DHEA acting specifically on tissues that possess the necessary receptors [20].

DHEA can be administered either orally or vaginally, with dosages varying accordingly. Several randomized controlled trials (RCTs) have demonstrated the efficacy of prasterone in treating GSM, particularly in reducing postmenopausal symptoms like dyspareunia and vaginal dryness [21,22]. When applied locally in varying doses (0.25%, 0.50%, 1.0%), prasterone improved vaginal cellular structure and lowered vaginal pH, providing relief from postmenopausal symptoms compared to placebo [13]. Daily DHEA use for two weeks, followed by a biweekly regimen [42] or a 12-week dosing schedule [21], was shown to have positive effects on vaginal atrophy, sexual function and other genitourinary symptoms. These improvements were reflected in the Female Sexual Function Index (FSFI), with notable enhancements in domains such as desire, arousal, lubrication, orgasm, satisfaction and pain [42].

Both the efficacy and safety of DHEA are evident when administered orally. A daily dose of 50 mg of DHEA over a year showed improvements in GSM compared to placebo without causing side effects [22]. Furthermore, the local application of DHEA, with its effects limited to the vagina, maintains serum sex hormone levels within the postmenopausal range, thereby avoiding the risk of endometrial stimulation [43]. Commonly reported side effects include vaginal discharge and abnormalities in cervical smears [44]. While DHEA is effective for treating dyspareunia and GSM symptoms, further research is required to assess its safety in women with hormone-dependent cancers.

### 4.5. Laser Therapy

In recent years, laser technology has emerged as a minimally invasive treatment option for pelvic floor dysfunctions, particularly for conditions such as genitourinary syndrome of menopause (GSM), vulvovaginal atrophy (VVA) and urinary incontinence [23]. The initial carbon dioxide (CO_2_) laser was soon followed by other variants, including the erbium: yttrium-aluminum-garnet (Er:YAG) and neodymium-doped: yttrium-aluminum-garnet (Nd:YAG) lasers. These laser systems deliver short pulses that impact the vaginal mucosa, promoting tissue regeneration [24]. Specifically, CO_2_ lasers induce collagen contraction through heat, leading to tissue remodeling, which stimulates the production of new collagen and elastic fibers, improving the extracellular matrix, epithelial integrity and vaginal moisture. These processes help restore vaginal mucosal elasticity and muscle tone [45].

A prospective cohort study treated 92 women with GSM and sexual discomfort using a fractionated CO_2_ laser. GSM severity and sexual quality of life were assessed using the Female Sexual Function Index (FSFI), Female Sexual Distress Scale (FSDS), Most Bothersome Symptoms (MBS) and Vaginal Health Index Score (VHIS). The results showed a significant reduction in bothersome symptoms and an improvement in sexual functionality and overall quality of life [45].

Furthermore, laser treatment has been shown to reduce the severity of dyspareunia [46] and alleviate symptoms such as itching, burning, dysuria and urinary incontinence [25,45,46]. The safety of gynecological laser treatments has been the subject of debate. In July 2018, the U.S. Food and Drug Administration (FDA) issued a warning about potential adverse events such as vaginal burns, scarring and pain [26]. However, several studies have provided evidence supporting the safety of laser therapy for postmenopausal women with GSM [27].

In 2020, a prospective observational study examined the safety and satisfaction levels of postmenopausal women treated with fractional CO_2_ lasers for VVA. The study found no severe complications and mild side effects were rare. Reported minor issues included one case of dizziness, minor bleeding, two cases of dysuria and one instance of symptoms resembling a vaginal infection, yet all participants reported high satisfaction levels [27].

A randomized, double-blind, placebo-controlled trial compared the efficacy of fractional CO_2_ laser therapy, estriol therapy and a combination of both in 45 women with VVA. The results demonstrated that both CO_2_ laser treatment alone and in combination with estriol significantly improved VVA symptoms [47]. Similarly, YAG laser therapy has also yielded positive outcomes, improving symptoms of VVA and urinary incontinence [48].Finally, we delve into new perspectives for the future. The use of ovarian tissue transplantation (OTCT) to delay menopause presents a promising alternative to traditional hormone replacement therapy by potentially restoring natural ovarian endocrine function. By reintroducing cryopreserved ovarian tissue during the menopausal transition, OTCT aims to extend endogenous estrogen production, thereby alleviating symptoms of menopause and reducing long-term health risks such as osteoporosis and cardiovascular disease. While the ability of OTCT to restore fertility is well-documented, its efficacy in mimicking the normal hormonal rhythm observed in reproductive years remains uncertain. Furthermore, repeated transplants may be necessary to achieve sustained ovarian function, but long-term data on the frequency and duration of graft success are limited. Current evidence is insufficient to confirm whether OTCT offers a superior safety and efficacy profile compared to HRT, according to few data derived from few patients [49,50,51,52].

On the other hand, new potential molecules are studied in rat models. A recent study focused on the pharmacotoxicological assessment of six innovative nanocapsule pharmaceutical formulations containing natural biomolecules for menopause treatment. The active molecules included nano diosgenin and glycyrrhizic acid, among others. In vitro toxicity tests on cultured cells and in vivo preclinical trials using a surgically induced menopause model in Wistar female rats were conducted. The micronucleus test indicated no genotoxicity for any of the tested formulas. Among the six nanocapsule formulas, the combination of nano diosgenin and glycyrrhizic acid showed promising hypoglycemic, hypolipidemic, hypouricemic and antioxidant properties, without significant safety concerns according to blood biochemical parameters [53].

## 5. Conclusions

The treatment of genitourinary syndrome of menopause (GSM) is multifaceted, given the variety of available therapeutic options. In the current landscape, the demand for personalized therapy is growing. Effective communication and counseling are crucial to inform women about the different treatment options. Physicians should encourage patients to make informed decisions based on their preferences and needs, as supported by the latest scientific evidence.

First-line treatment for GSM includes lubricants and moisturizers, which are generally recommended for mild to moderate symptoms to provide short-term relief. However, these non-hormonal treatments address symptoms without treating the underlying cause or preventing the progression of GSM. When symptoms persist or are moderate to severe, hormonal therapy, either topical or systemic, is suggested as a second-line treatment, depending on the patient’s needs and preferences. Research shows that topical hormone treatments carry fewer side effects than systemic options. For example, vaginal estrogen is suitable for women with urogenital atrophy when systemic hormones are contraindicated, as estrogen levels after vaginal application remain within normal postmenopausal ranges. DHEA is a hormone therapy option that does not involve estrogen, making it a suitable choice for women with symptomatic GSM, particularly those experiencing dyspareunia and sexual discomfort. For patients who do not respond well to vaginal therapy or cannot use hormone treatments, alternatives like oral ospemifene or laser therapy may be considered. The safety of hormonal therapy for GSM in women with a history of cancer or who are at risk of cancer remains a contentious issue. Medical societies such as ACOG and NAMS have expressed mixed views on the use of local estrogen therapy in these populations. The safety of these hormonal therapies in cancer survivors is still unclear and recommendations remain controversial. Current studies on managing GSM in women with a cancer history or risk are limited. A randomized trial involving 16,608 postmenopausal women with GSM treated with estrogen plus progestin compared to placebo found no increased risk of endometrial cancer, a conclusion supported by multiple studies [54]. For breast cancer survivors, non-hormonal measures appear to be safe [7,8,43]. Some researchers suggest that low-dose vaginal estrogens may be considered [54], but long-term controlled clinical trials are needed to confirm their safety in women with a history of cancer. The literature is inconclusive regarding the cancer risk associated with vaginal DHEA and vaginal estrogen, making it difficult to recommend one over the other for cancer survivors. Opinions on the cancer risk of ospemifene also differ [55]. As a Selective Estrogen Receptor Modulator (SERM), ospemifene acts similarly to tamoxifen in breast and endometrial tissues, though with less potency and is thought to have more beneficial than harmful effects.

In conclusion, GSM reflects the negative impact of declining estrogen levels during menopause, affecting not only vaginal health but also significantly reducing women’s quality of life. These symptoms are often underdiagnosed and undertreated. Clinicians must provide individualized treatment options based on a woman’s clinical history and the range of available therapies. More research is needed to expand the evidence base and this review highlights the most relevant studies from leading scientific databases.

## Figures and Tables

**Table 1:** Summary of treatment options for genitourinary syndrome of menopause (GSM).

Treatment	Mechanism of Action	Effects	Side Effects	Application Method
Ospemifene	Selective estrogen receptor modulator	Improves vaginal atrophy symptoms	Hot flushes, urinary tract infections, headache	Oral
DHEA (dehydroepiandrosterone)	Steroid hormone conversion of cholesterol into androgens and estrogens	Improves dyspareunia and vaginal dryness, increases well-being in sexual life	Vaginal discharge, abnormalities in the cervical smear	Oral/Vaginal
Laser Therapy (CO_2_, Er:YAG, Nd:YAG)	Tissue regeneration via heat-induced collagen contraction	Improves GSM, VVA, urinary incontinence, restores vaginal mucosa elasticity	Potential vaginal burns, scarring, pain	Local
Lubricants/ Moisturizers	Symptom relief	Provides short-term relief for mild to moderate symptoms	Dependent on the specific product used	Topical
Hormonal Therapy (topical/systemic)	Restoration of hormonal balance	Improves moderate to severe GSM	Dependent on hormone type and specific patient	Topical/Systemic

**Table 2:** Characteristics and data extracted of included studies about principal treatment options for genitourinary syndrome of menopause (GSM).

Study	Years	Type of Study	n. pts	Treatment	Duration of Treatment	Outcomes	Results
Schiavi MC [9]	2019	Retrospective	98	hyaluronic acid, chondroitin sulfate, curcumin and quercetin	6 months	The efficacy of orally administered combination of hyaluronic acid, chondroitin sulfate, curcumin and quercetin for the prevention of postcoital recurrent urinary tract infection	Reduction of episodes of dysuria and the number of urinations Improvement in pelvic pain, urinary urgency, female sexual function index, female sexual distress scale
Herbenick D [10]	2011	RCT	2453	water- or silicone-based lubricants	5 weeks	Used lubricant during partnered and solo sexual activities Sexual pleasure and satisfaction Evaluate the extent to which lubricant use was associated with subsequent genital symptoms	Water-based lubricants were associated with fewer genital symptoms compared with silicone-based lubricants Water-based lubricant use was associated with higher ratings of sexual pleasure and satisfaction for penile–anal sex as compared with no lubricant use
Chen J [11]	2013	RCT	144	hyaluronic acid vaginal gel vs. estriol cream	30 days	Evaluate the efficacy and safety of hyaluronic acid vaginal gel to treat vaginal dryness compared with estriol cream in postmenopausal women	Both hyaluronic acid vaginal gel and estriol cream can significantly improve the clinical symptoms of vaginal dryness in postmenopausal women
Bachmann G [12]	2009	RCT	423	conjugated estrogens cream vs. placebo	12 weeks, followed by open-label treatment with estrogen cream for 40 weeks	Evaluate the efficacy and safety of low-dose conjugated estrogen cream for treatment of atrophic vaginitis	Improvements in vaginal maturation index, in vaginal pH Reduction of bothersome symptoms such as dyspareunia
Griesser H [13]	2012	RCT	436	pessaries with 0.2 mg estriol vs. pessariess with 0.03 mg estriol vs. placebo	12 weeks	The efficacy of estriol containing pessaries compared to placebo in the treatment of vaginal atrophy	Increase in vaginal maturation index compared to placebo Decrease in vaginal pH compared to placebo Reduction in most bothersome symptom intensity compared to placebo
Hosseinzadeh P [14]	2015	RCT	160	estradiol vaginal tablet vs. vaginal estrogen cream	12 weeks	Evaluate the improvement of symptoms of atrophic vaginitis	Estrogen vaginal tablet is an appropriate medication for the treatment of atrophic vaginitis, which is as effective as vaginal estrogen creams and is more user-friendly
Lose G [15]	2000	RCT	251	estradiol-releasing ring vs. estriol pessaries 0.5 mg	24 weeks	Subjective scores of urgency, frequency, nocturia, dysuria, stress incontinence and urge incontinence	Two treatments were equally efficacious in alleviating urinary urgency, urge incontinence, stress incontinence and nocturia
Barnabei MV [16]	2005	RCT	16,608	one tablet containing 0.625 mg conjugated equine estrogens plus 2.5 mg medroxyprogesterone acetate vs. placebo	followed for a mean of 5.6 years	Estimate the effects of estrogen plus progestin therapy on menopausal symptoms, vaginal bleeding, gynecologic surgery rates and treatment-related adverse effects in postmenopausal women	Estrogen plus progestin relieved some menopausal symptoms, such as vasomotor symptoms and vaginal or genital dryness, but contributed to treatment-related effects, such as bleeding, breast tenderness and an increased likelihood of gynecologic surgery
Alvisi S [17]	2017	N/A	32	ospemifene vs. no hormone	N/A	To evaluate the effects of ospemifene on histological parameters, glycogen content, proliferation and estrogen receptor a expression of vaginal epithelium in postmenopausal women	Intake of ospemifene is associated with an increased maturation, and estrogen receptor a expression of the vaginal mucosa
Schiavi MC [18]	2019	Retrospective	81	ospemifene	12 weeks	Evaluate the effectiveness and safety of ospemifene in the improvement of urgency component in women affected by mixed urinary incontinence who underwent surgery with mid-urethral sling	Improvement in the average number of urinations, episodes of urgent urination/24 h, urgent urinary incontinence and in nocturia events
Labrie F [19]	2018	RCT	482	intravaginal dehydroepiandrosterone vs. placebo	12 weeks	Local beneficial effects of intravaginal dehydroepiandrosterone on moderate to severe dyspareunia or pain at sexual activity, percentage of parabasal cells, percentage or superficial cells, vaginal pH	Decrease the percentage of parabasal cells, pain at sexual activity decreased and vaginal pH Increase the percentage of superficial cells
Panjari M [20]	2009	RCT	93	dehydroepiandrosterone vs. placebo	52 weeks	Total satisfying sexual events and the change in the Sabbatsberg Sexual Self-Rating Scale total score	No differences between the dehydroepiandrosterone and placebo
Bouchard C [21]	2015	RCT	450	intravaginal administration of 0.25% dehydroepiandrosterone vs. 0.50% dehydroepiandrosterone vs. placebo	12 weeks	Analyzes the effect of a reduced dosing regimen of dehydroepiandrosterone on most bothersome symptoms	Decrease in percentage of parabasal cells, vaginal pH, vaginal dryness and dyspareunia Increase in percentage of superficial cells
Portman DJ [22]	2015	RCT	422	intravaginal dehydroepiandrosterone vs. placebo	52 weeks	Effects of intravaginal dehydroepiandrosterone on the endometrium in postmenopausal women	Endometrial atrophy was found in all women treated with intravaginal dehydroepiandrosterone
Di Donato V [23]	2022	Prospective	92	fractional CO_2_ laser Lumenis AcuPulse	3 months	Improvement of vaginal health and signs and symptoms associated with GSM	Improvement in vaginal itching, post-coital vaginal bleeding, vaginal dryness, dyspareunia, dysuria and vaginal PH Higher Vaginal Health Index Score
Paraiso M F R [24]	2020	RCT	62	fractionated CO_2_ vaginal laser therapy vs. estrogen vaginal cream.	6 months	Evaluation of vaginal atrophy, quality of life symptoms, assessment of sexual function and urinary symptoms	85.8% of laser participants rated their improvement as “better or much better” and 78.5% reported being either “satisfied or very satisfied” compared to 70% and 73.3% in the estrogen group
Di Donato V [25]	2020	Prospective	53	fractionated CO_2_ vaginal laser therapy	N/A	Evaluate the safety of and patient satisfaction with fractional CO_2_ laser for the treatment of vulvovaginal atrophy	89.7 % of patients would highly recommend the procedure 94.9 % would be ready to repeat the procedure to maintain results
Cruz VL [26]	2018	RCT	45	fractionated CO_2_ vaginal laser therapy vs. local estrogen therapy vs. the combination of both treatments	20 weeks	Evaluate efficacy of fractional CO_2_ vaginal laser treatment and compare it to local estrogen therapy and the combination of both treatments in the treatment of vulvovaginal atrophy	CO_2_ vaginal laser alone or in combination with topical estriol is a good treatment option for VVA symptoms
Blaganje M [27]	2018	RCT	114	fractionated CO_2_ vaginal laser therapy vs. placebo	3 months	Evaluate the efficacy and safety of non-ablative Er:YAG laser therapy in the treatment of stress urinary incontinence and improvement of sexual gratification in parous women	Improvement in the impact of stress urinary incontinence symptoms on quality of life and sexual function in premenopausal parous women

## Data Availability

All data are provided within the text.
